# Supplementary material for: Full Length Transcriptome Highlights the Coordination of Plastid Transcript Processing
Source: Int J Mol Sci. 2021 Oct 19;22(20):11297. doi: 10.3390/ijms222011297 (PMC8537030; doi:10.3390/ijms222011297)
Supplement: Supplementary file 1 [file ijms-22-11297-s001.zip › ijms-1379195-supplementary.pdf]

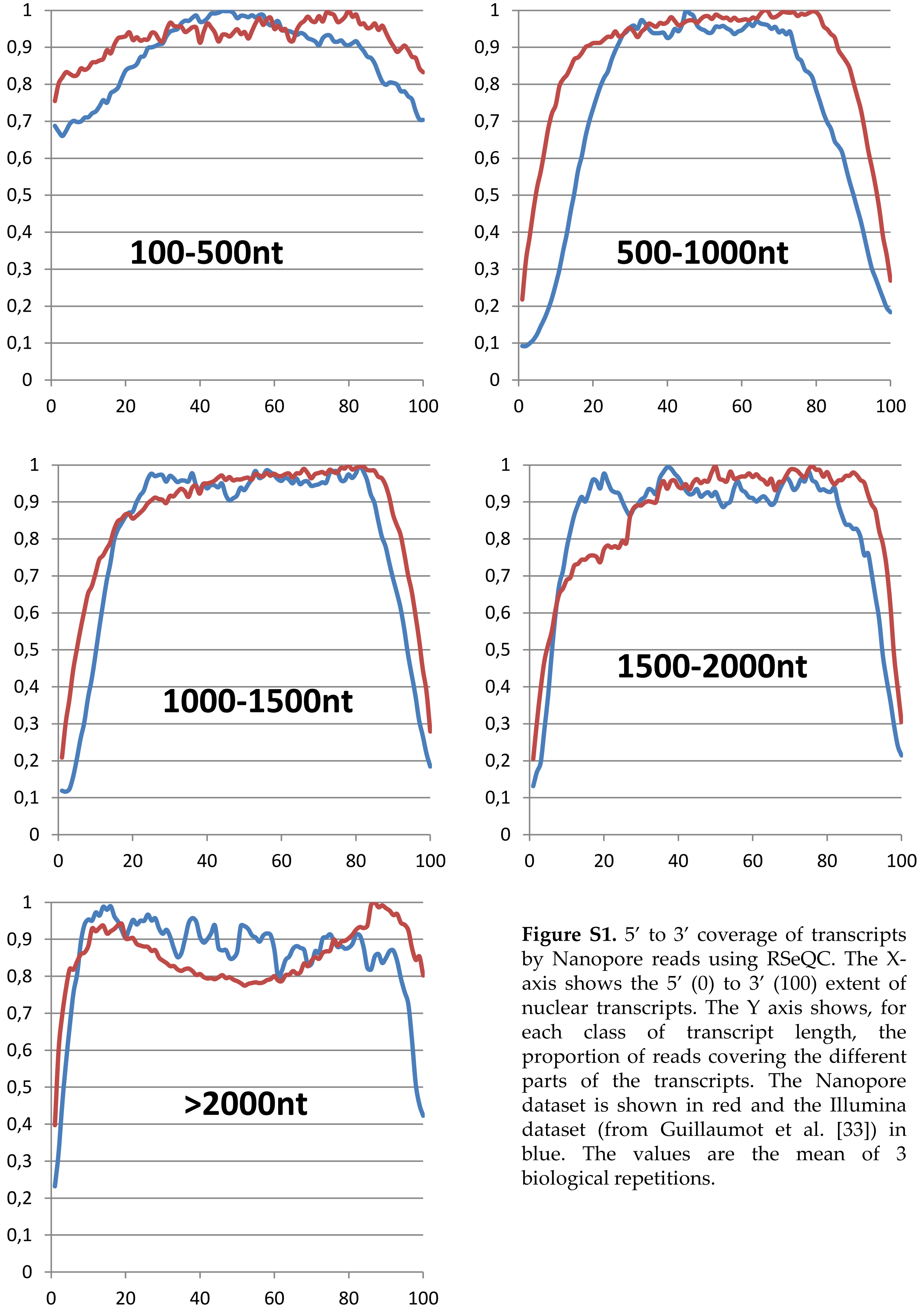

Figure S1. 5′ to 3′ coverage of transcripts by Nanopore reads using RSeQC. The X-axis shows the 5′ (0) to 3′ (100) extent of nuclear transcripts. The Y axis shows, for each class of transcript length, the proportion of reads covering the different parts of the transcripts. The Nanopore dataset is shown in red and the Illumina dataset (from Guillaumot et al. [33]) in blue. The values are the mean of 3 biological repetitions.

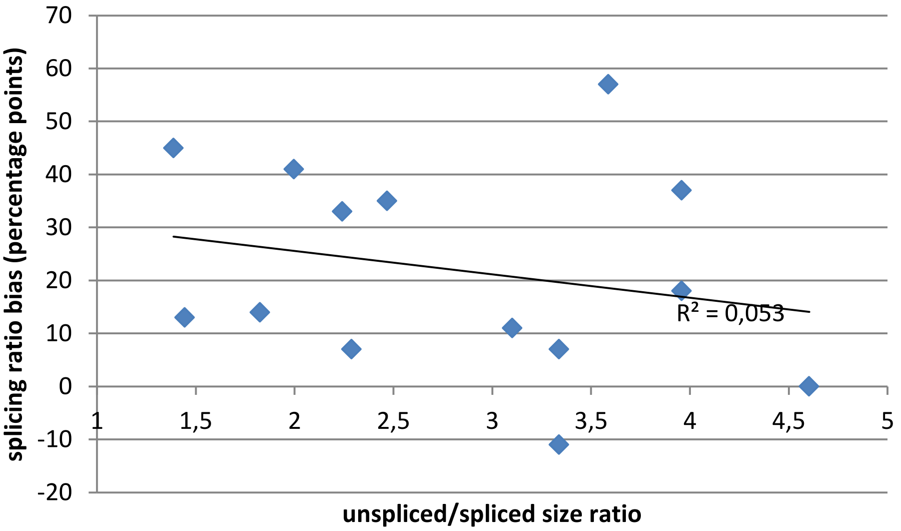

P-value=0.4286

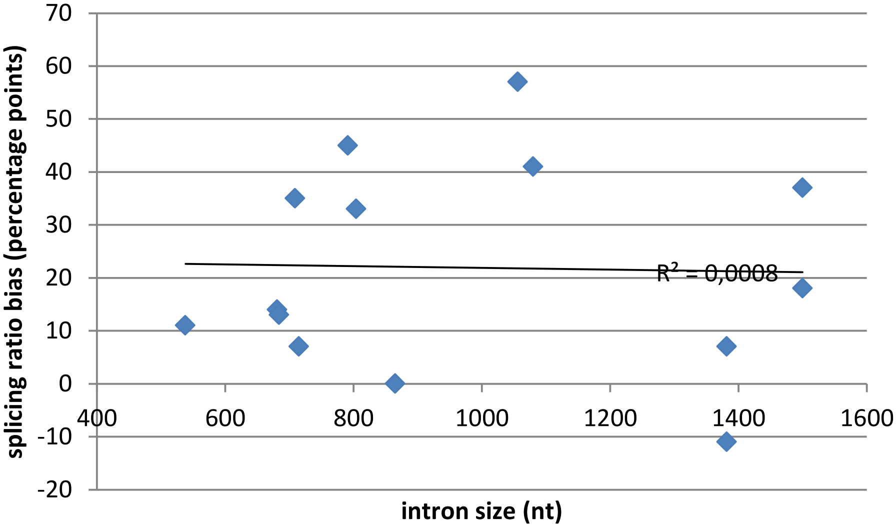

P-value=0.9239

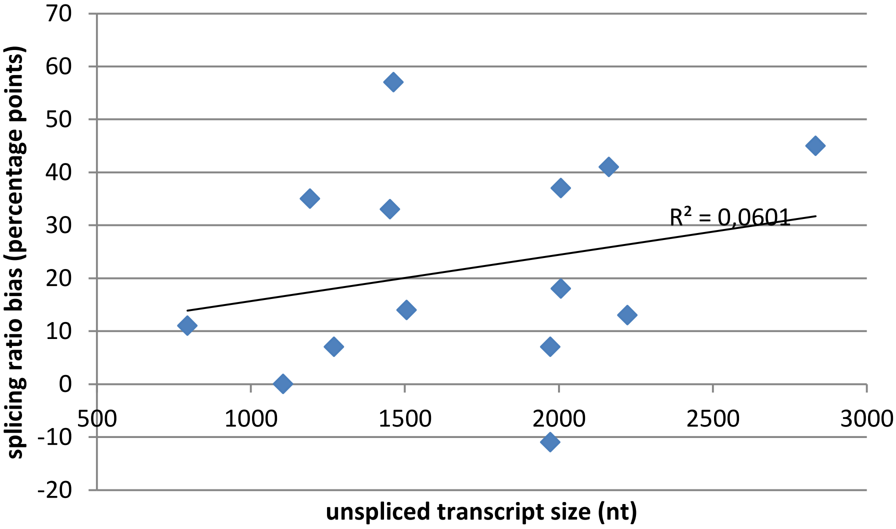

P-value=0.3984
